# In this month’s Bulletin 

**DOI:** 10.2471/BLT.23.001123

**Published:** 2023-11-01

In editorials this month, Muhammad Naveed Noor et al. (682) argue the case for a classification of the private health sector. Sadia Shakoor and Sabine Dittrich (683) point out that better diagnostic tools are needed to distinguish typhoid from other causes of acute febrile illness.

Fid Thomson (686–687) reports on the potential of affordable solar power to transform health service delivery in facilities with no electricity. Christian Owoo talks to Gary Humphreys (688–689) about the clinical challenges faced during the COVID-19 pandemic and the importance of adapting guidance to local conditions.

## Bangladesh, Bhutan, Brunei Darussalam, Cambodia, China, Democratic People's Republic of Korea, India, Indonesia, Lao People's Democratic Republic, Malaysia, Maldives, Myanmar, Nepal, Philippines, Sri Lanka, Thailand, Timor-Leste, Viet Nam

### Monitoring child growth and nutrition

Remco Peters et al. (690–706) review national surveillance programmes.

## Somalia

### Revising the essential package of health services

Mohamed A Jama et al. (738–742) align stakeholders on improved service delivery.

## Global

### Assessing risk of SARS-CoV-2 variants 

Homa Attar Cohen et al. (707–716) describe ongoing genomic surveillance efforts

### Public health and social measures during emergencies

Ramona Ludolph et al. (717–722) present a research agenda.

### Assisted vaginal birth

Ana Pilar Betrán et al. (723–729) detail the research needed to improve outcomes for mothers and babies. 

### Target product profiles for tuberculosis 

Ankur Gupta-Wright et al. (730–737) list the desired characteristics of new tests.

### Defining burnout

Renzo Bianchi and Irvin Sam Schonfeld (743–745) reexamine the basis of original definitions.

